# Tracking longitudinal genetic changes of circulating tumor DNA (ctDNA) in advanced Lung adenocarcinoma treated with chemotherapy

**DOI:** 10.1186/s12967-019-2087-9

**Published:** 2019-10-10

**Authors:** Xiaohong Han, Ying Han, Qiaoyun Tan, Yu Huang, Jianliang Yang, Sheng Yang, Xiaohui He, Shengyu Zhou, Yan Song, Jinping Pi, Lijie Zuo, Jiarui Yao, Di Wu, Zhishang Zhang, Yuankai Shi

**Affiliations:** 1 Department of Medical Oncology, National Cancer Center/National Clinical Research Center for Cancer/Cancer Hospital, Chinese Academy of Medical Sciences & Peking Union Medical College, Beijing Key Laboratory of Clinical Study on Anticancer Molecular Targeted Drugs, Beijing, China; 2 Department of Clinical Laboratory, National Cancer Center/National Clinical Research Center for Cancer/Cancer Hospital, Chinese Academy of Medical Sciences & Peking Union Medical College, Beijing, China; 3 Beijing Chaoyang Sanhuan Cancer Hospital, Beijing, China

**Keywords:** CtDNA, Efficacy, Chemotherapy, Lung adenocarcinoma

## Abstract

**Introduction:**

Pemetrexed combined with platinum complexes can be used as first-line treatment for advanced non-squamous non-small cell lung cancer (NSCLC), however, the efficacy and safety is varying from individuals. There is a need to better understand the genetic variations associated with platinum response.

**Materials and Methods:**

We performed next-generation sequencing (NGS) based on BGI Oseq-ctDNA panel to analyze 98 longitudinal plasma samples from 32 lung adenocarcinoma patients during platinum-based chemotherapy, and a bioinformatic pipeline was developed to detect point mutations.

**Results:**

We found that mutation burden was decreased after chemotherapy, which reflected chemotherapy sensitivity, especially the frequency of C>G and C>A substitutions. Moreover, neoplastic cells carrying a specific set of somatic mutations, such as *EGFR(L858R)*, *KRAS* (p.G12C) were obviously correlated with platinum treatment. In addition, the MAPK pathway was found to have a pivotal role in NSCLC and platinum based response. Finally, we found that smokers benefit less from platinum-based chemotherapy.

**Conclusions:**

Collectively, this work described the dynamic changes of ctDNA mutation status during platinum-based treatment, which may contribute to advanced lung adenocarcinoma patients stratification and precision treatment.

## Introduction

Lung cancer, of which the major subtype is non-small cell lung cancer (NSCLC), is one of the leading causes of cancer-related deaths worldwide [1]. The large scales of patients with NSCLC are diagnosed with metastatic disease, which is generally fatal and experiences a low 5-year survival rate (≤ 5%) when diagnosed with stage IIIB or IV [2]. More than 80% of NSCLC cases are adenocarcinoma subtype, whose incidence rate has steadily increased over the past decades [3]. Platinum-based chemotherapy was recommended as first-line chemotherapy regimen for advanced NSCLC in National Comprehensive Cancer Network (NCCN) guideline, especially, those without epidermal growth factor receptor (EGFR) mutations or anaplastic lymphoma kinase (ALK) rearrangement and proto-oncogene tyrosine-protein kinase ROS (ROS1) translocations [4]. However, clinical outcomes is varying from individuals, and no widely applicable biomarkers have been successfully applied to daily clinical practice. Due to the little information about biomarkers to evaluate the chemotherapy response, it is clinically important to find out novel predictive markers for treatment response and survival after platinum-based chemotherapy in patients with NSCLC. Currently, development of next-generation sequencing (NGS) technology and genotyping has offered promising prospects on the molecular pathology of NSCLC.

The repeat tissue biopsy can potentially provide prognostic information on chemotherapy efficacy but is limited to longitudinal monitoring for its invasive manipulation [5]. Furthermore, it may fail to reflect the intra- and inter-tumor genetic heterogeneity [6, 7]. Compared with tissue biopsy, blood is easier to obtain and less expensive. Besides, it can deliver a more comprehensive genomic profiling because tumor either in first-site or metastases can shed genomic DNA information to the bloodstream [8, 9]. Liquid biopsy via circulating tumor DNA (ctDNA) in blood provides an attractive alternative for long terms evaluation and prediction for lung cancer patients and ctDNA level may provide a more comprehensive picture of the lung cancer, because markers spreading in the blood may contain cancer-associated materials from many diseases site in the body organs.

An increased understanding about ctDNA as predictor and biomarker for disease response and survival in NSCLC patients has come forth in recent years. Some studies showed the ctDNA concentration is associated with poor prognostic results [10, 11], while other studies assessed the predictive and prognostic value of cfDNA concentration or EGFR mutation in NSCLC patients treated with chemotherapy [12], but these studies are limited to obtain a few genes, not reflecting the full spectrum of mutations emerging during the treatment. In addition, there is a lack of clinical information on prediction of efficacy for advanced NSCLC patients after chemotherapy, especially through dynamic monitoring.

Here we assessed the genetic dynamics changes in ctDNA before and during chemotherapy treatment, a targeted sequencing panel Oseq-ctDNA based on the Illumina platform was used with a customized bioinformatics pipeline to identify novel responsiveness associated biomarkers and potential actionable targets in advanced lung adenocarcinoma.

## Materials and methods

### Study design and patients selection

We conducted a prospective study with 32 consecutive patients recruited who was histologically confirmed advanced or metastatic lung adenocarcinoma between April 2015 and June 2016, they all received platinum based chemotherapy as the first-line treatment in Cancer Hospital, Chinese Academy of Medical Sciences. Baseline blood samples were taken within a week before the first dose of chemotherapy, serial follow-up samples were obtained after each treatment cycle, the detailed clinicopathological information and blood draw time points for each patient were shown in Additional file 1: Table S1 and Additional file 2: Table S2. Clinical information was collected from electronic medical record system or telephone follow-ups. Clinical staging was determined using chest computed tomography, brain magnetic resonance imaging, and 18F-fluorodeoxyglucose positron emission tomography based on the 7th lung cancer TNM classification and staging system. Response to treatment was examined by computed tomography every two cycles and evaluated according to the Response Evaluation Criteria in Solid Tumors (RECIST) 1.1 as complete response (CR), partial response (PR), stable disease (SD), or progressive disease (PD) [13]. The study was approved by the Independent Review Board (IRB) of Cancer Hospital, Chinese Academy of Medical Sciences and BGI Genomics. (CH-BMS-018, BGI-IRB18035).

### cfDNA extraction and sequencing

The genomic DNAs were extracted form plasma samples and matched germline DNA from white blood cells using Tiangen genomic DNA extraction kit (DP318) according to the manufacturer’s standard protocol. 1 µg of DNA from each sample was used for library construction. At first, genomic DNA fragmentation was performed using an ultrasonoscope (Covaris E220; Covaris, Massachusetts, USA) to generate fragments with a peak of 250 bps with intensity H, time 5 min and cycle 6. After purification with AMPure beads (Beckman Coulter, Brea, USA), the DNAs were treated with T4 DNA polymerase, T4 polynucleotide kinase and the Klenow fragment of *E. coli* DNA polymerase for 30 min on 20 °C in a thermocycler to generate blunt phosphorylated DNAs. A 4-cycle polymerase chain reaction (PCR) was performed with primers containing unique 8 bp-index sequence to mark samples and purify. The libraries which were the purified PCR products were quantified by Bioanalyzer 2100 instrument (Agilent Technologies, Palo Alto, USA) and Qubit 2.0 (Inverogen, USA). DNA target enrichment was performed on a custom sequence capture-array (Roche, Bael, Switzerland). The pooled library with a combined mass about 5 μg was used in the target region to capture hybridization. After 72 h hybridization at 42 °C, the DNA fragments bound to the array were washed and eluted with 125 mM sodium hydroxide. The elution product was purified using QIAquick PCR Purification Kit (Qiagen, Hilden, Germany) after following a 15-cycle ligation-mediated PCR to enrich the captured DNAs. The size and quantity of the captured library were assessed by Bioanalyzer 2100 instrument and Qubit (Invitrogen) and the enrichment of target region was assayed by QRT-PCR. Genomic DNA sequencing was performed on a HiSeq 2500 sequencing system (Illumina, San Diego, CA) with 2× 101-bp, paired-end reads were 100-bp and single index read 8-bp using SBS Kit v4 chemistry on a HiSeq 2500 loaded onto an Illumina cBot instrument at 50 pmol/L for cluster generation according to the manufacturer’s instructions.

### Whole-exome sequencing

Whole-exome sequencing was performed on genomic DNAs from two tumors and matched blood samples. The SureSelectXT Human All Exon V5 capture library (Agilent) for 50 Mb of exonic regions was used to capture the exonic DNA. According to the manufacturer’s instructions, we constructed the sequence library with the SureSelectXT Target Enrichment System for Illumina Paired-End Sequencing Library kit (Agilent). Then DNA sequencing of 100-bp paired-end reads were performed using the Illumina HiSeq4000 sequencer.

### Sequencing data processing

Sequencing data was filtered with SOAPnuke (v1.5.0; https://github.com/BGI-flexlab/SOAPnuke) to remove sequencing adapters and low quality reads. We used BWA (v0.5.9; http://bio-bwa.sourceforge.net/) with default parameters to align high-quality reads to the NCBI human reference genome (hg19). Picard (v1.54; http://broadinstitute.github.io/picard/) and Genome Analysis Toolkit (v1.0.6076, GATK IndelRealigner; https://software.broadinstitute.org/gatk/) [14] were used to mark duplicates reads and improve alignment accuracy, respectively.

The potential somatic single-nucleotide variants (SNVs) were called by two software based on paired-alignment files (tumor and normal bam).One was MutTect (http://archive.broadinstitute.org/cancer/cga/mutect) using default parameters(–initial_tumor_lod < 4.0 > , Initial LOD threshold for calling tumor variant–tumor_lod < 6.3 > ,LOD threshold for calling tumor variant–normal_lod < 2.2 > ,LOD threshold for calling normal non-germline–dbsnp_normal_lod < 5.5 > ,LOD threshold for calling normal non-variant at dbsnp sites –pir_median_threshold < 10.0 > ,threshold for clustered read position artifact median –pir_mad _threshold < 3.0 > , threshold for clustered read position artifact MAD–max_alt_alleles_in_normal_count < 1>,threshold for maximum alternate allele counts in normal–max_alt_alleles_in_normal_qscore_sum < 20 > ,threshold for maximum alternate allele quality score sum in normal–max_alt_allele _in_normal _fraction < 0.03 > ,threshold for maximum alternate allele fraction in normal–power_constant_qscore < 30 > , Phred scale quality score constant to use in power calculations), and the other was VarScan (https://github.com/dkoboldt/varscan) with the following parameters: –min-coverage-normal 10 –min-coverage-tumor 14 –min-var-freq 0.001 –somatic-*p* value 0.05.

### Bioinformatics pipeline

We first got the original bam (Bam File A). Considering the fact that the length distribution of cfDNA fragments have a dominant peak at ~ 167 bp, whereas the distribution peak of ctDNA fragments was ~ 133 bp. And there is positive correlation between the proportion of short DNA (below 150 bp) and the amount of tumor DNA in the plasma of cancer cases [15]. We reconstructed the bam file by selecting the reads whose insert size was smaller than 150 bp, designated as remoulded bam (Bam File B). Following the method calling SNVs showed in Materials and methods section ‘Sequencing data processing**’** section, we detected potential somatic SNVs based on paired alignment files (tumor and normal bam).

Here we developed a software to filter the initial point mutations by following conditions: (1) In tumor, the mutant site should have ≥ 4 mutant reads with ≥ 1 reads on each strand. In normal, the mutant site can only have ≤ 2 mutant reads with the normal VAF < 0.01 and isn’t within the dbSNP131 database. They are required with at least 40-fold coverage in normal and tumor. The mutations with supported reads ≥ 1 in normal and present in ExAC with AF ≥ 0.001 or with supported reads ≥ 2 in normal but not present in COSMIC database were removed. (2)The number of mutant reads covered this site must be an outlier in a given window around it, and less than 3 smaller insert and deletion in site <=10 bp from a predicted SNV, but 0 string mismatch base pairs in reads located on this mutant site. In addition, mismatching bases can`t enrich in this region which can determine by our software. Here we select 11 bp region around a predicted SNV as a window to analysis. /93) A power which represents the probability of a mutation detected in the plasma was calculated via our software, and those mutations with detected power more than 80 were kept. /94) A posterior probability of a predicted SNV based on TCGA database was also evaluated by our software, and those mutations with detected posterior probability more than 0.8 were kept. /95) Choose the PASS type mutations via the published perl script (https://github.com/ucscCancer/fpfilter-tool) with the default parameter.

Finally, all the outcomes were checked one by one whether it is caused by nearby misaligned small insertion and deletion events or sequence similarity in the genome, leading to misplacement of reads in the original bam. Then we can get all the mutations with more confidence. All those mutations were annotated by ANNOVAR(http://annovar.openbioinformatics.org/en/latest/) [16].

### Mutational processing in plasma

Mutation spectrum analysis was based on the six possible base changes pre- or post- chemotherapy. Then we displayed the proportion of 96 possible mutation types with the R package lwlegopt (https://github.com/BGI-LuoWen/lwlegopt). To determine the dynamic of mutational processes in plasma, all point mutations pre- chemotherapy were analyzed using MutationalPatterns R package [17] based on the 30 of COSMIC signatures (https://cancer.sanger.ac.uk/cosmic/signatures).

### Statistical analyses

All statistical test were performed in R. In this study, Student’s t-test was used for determining significance of point mutations. Variant allele frequency of mutations was usually tested with a non-parametric Mann–Whitney U test, as well as gene expression between normal and tumor samples, but not for the variant allele frequency of mutations between smoker and nonsmoker which was tested with one-way ANOVA test. In addition, Fisher’s exact test and Cochran-Mantel–Haenszel Chi Squared test were also used in non-silent SNVs, clinical pathological features and Additional file 6: Table S4. And finally, the log-rank test was used to perform overall survival (OS) and progression-free survival (PFS) analysis. For all statistical test used, we assumed that there is independence between data. Box plots showed median values and middle quartile.

## Results

### Data cohort and analytic approaches

**Fig. 1:**
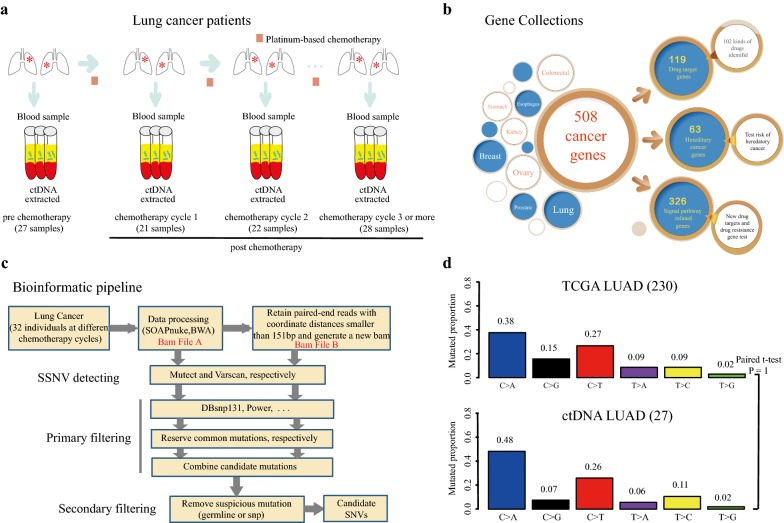
The study methodology overview and verification. **a** Cell-free DNA (cfDNA) was extracted from blood samples which were collected from 32 lung cancer patients during each chemotherapy cycle. Square with light scarlet referred to the first-line treatment: platinum-based chemotherapy. **b** Gene collections of Oseq-ctDNA panel which involves 508 cancer-related genes. **c** Bioinformatic pipeline for candidate mutations detecting. **d** Landscape of genetic alterations between 27 pre-chemotherapy samples and TCGA cohort. A bar represented one of six substitution subtypes with different colors. The y-axis represented mutated proportion

In this study, we collected 98 plasma samples from 32 patients during chemotherapy (Fig. 1a). All patients were histologically confirmed advanced (28.1% at stage III and 71.9% at stage IV) lung adenocarcinoma in China (Additional file 1: Table S1 and Additional file 2: Table S2). Of these, 65.6% were men and half of them had malignant pleural effusion. Patients were diagnosed with a median age 60.5 years (range, 43-75 years), and 59.4% of them were smokers. Platinum-based chemotherapy was the first-line treatment for them, 93.7% were treated with pemetrexed combined platinum (53.1% cisplatin and 40.6% carboplatin), and 12 patients received TKI treatment after chemotherapy failure.

To analyze 28 pre-chemotherapy and 68 post-chemotherapy plasma DNAs, we performed a next-generation sequencing (NGS) based on BGI Oseq-ctDNA, a panel that involves 508 cancer-related genes (Fig. 1b; Additional file 3: Table S3).The average coverage of target regions were approximately 274 × for normal control, but 991x for tumors (Additional file 4: Figure S1A). We followed the pipeline to detect somatic mutations (Fig. 1c). Through this pipeline, many candidate point mutations were observed with a mean variant allele frequency (VAF) more than 1% which previous report was counted [18] (Additional file 4: Figure S1B). The genetic alteration landscape of the target coding region across those 27 samples pre- chemotherapy were very similar to TCGA-exome cohort (Fig. 1d).

To further evaluate those point mutations, we performed a screening for two tissue samples pre- chemotherapy which were randomly selected from the 32 patients. Whole exome sequencing was used to analyze these two tissues with an average coverage of 200x. 27 out of 101 (26.7%) alterations were identified in both plasma samples and tissue samples (Additional file 4: Figure S1C, Additional file 6: Table S4).

### Mutation burden decreases after platinum treatment

**Fig. 2:**
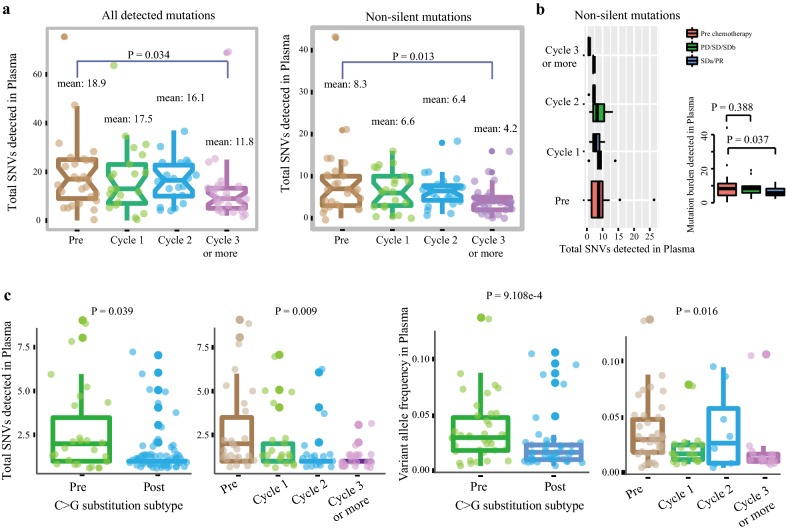
Total SNVs detected in plasma. **a** The distribution of all detected SNVs and non-silent mutations during platinum treatment was shown in the panel. The y-axis represented the mutation number. Each box represented each group and each dot was on behalf of one sample. **b** A comparison of non-silent mutations between platinum sensitive (SDa/PR) and platinum insensitive (PD/SD/SDb). The x-axis represented the mutation number detected in plasma and the y-axis represented the groups. **c** The distribution of C>G substitution subtype during chemotherapy. The y-axis represented the number of C>G substitution subtype in the two panel on the left with each dot representing one sample, whereas y-axis on behalf of the VAFs in the two panel on the right with each dot representing one mutation

We identified a total of 1559 point mutations across 98 plasma samples, including 637 non-silents, 262 silents and 660 non-coding mutations. Among the 98 tumor samples, 98% (96/98) carried one or more point mutations during chemotherapy (range, 1–75 mutations), showing a diversity of mutation detection rate of patients during chemotherapy (Additional file 5: Figure S2A). Simultaneously, we found that whole mutation burden and non-silent mutation burden both gradually decreased following platinum-based chemotherapy,with p value 0.034 and 0.013, respectively (Fig. 2a). Moreover, patients with better response to platinum carried less non-silent mutations after chemotherapy compared to those insensitive patients (Fig. 2b). In addition, non-silent SNVs at baseline was higher in patients with response (PR or SDa) compared to non- responders (SD or PD), objective response rate was also greater in patients with higher non-silent SNVs (> median, 7 mutations) compared with low non-silent SNVs (≤ median), although not reaching statistical significance likely owing to small numbers (Additional file 5: Figure S2B).

When we explore the different spectra following chemotherapy, we found that C>G mutations were significantly decreased during platinum treatment, no matter total SNVs (p = 0.039; Fig. 2c) or VAF (p < 0.01; Fig. 2c), especially in patients with objective response (PR or SDa) (p = 0.029; Additional file 5: Figure S2C), suggesting a significant correction between C>G mutations and platinum-based response. Furthermore, C>A transversion, which was reported to be correlated with smoking status [19–21], the mutation levels were generally low post chemotherapy (p = 0.003; Additional file 5: Figure S2D), especially in non-smokers(Additional file 5: Figure S2E).

### Mutational signatures in LUAD during chemotherapy

**Fig. 3:**
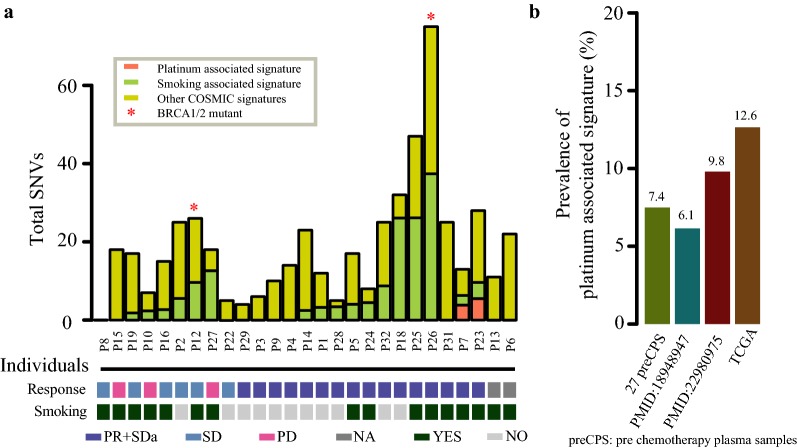
Signatures of mutational processes in 27 pre-chemotherapy plasma samples. Barplot showed the prevalence of platinum associated signature for four independent cohorts on the left. Total SNVs contributed to platinum associated signature (coral1) and smoking associated signature (darkolivegreen3) was shown in the panel on the right. Vertical axis represented the samples. *Represented BRCA1/2 mutant. Those samples with sensitive response were indicated in darkslateblue and smokers were indicated in darkslategrey

We analyzed point mutations of 27 plasma pre-treatment samples, the result indicated that almost 63% (17/27) patients characterized by smoking associated signatures (COSMIC Signatures 4, 16 and 29) [22], of which 64.7% (11/17) were smokers (Fig. 3a), showing that smoking associated signatures were ubiquitous signatures in LUAD [23]. However, there is no relationship between smoking associated signatures and platinum response (Additional file 6: Table S4). Interestingly, we noticed that 2 out of 27 (7.4%) patients displayed a prominent contribution of platinum associated signature (COSMIC Signatures 3) with platinum sensitivity. These findings were further validated in three independent cohorts including TCGA with more than 6.1% cases were characterized by platinum associated signatures (Fig. 3b). The results indicated that mutation spectra and signatures could be factors influencing platinum response [24].

### Gene silencing and benefit during chemotherapy

**Fig. 4:**
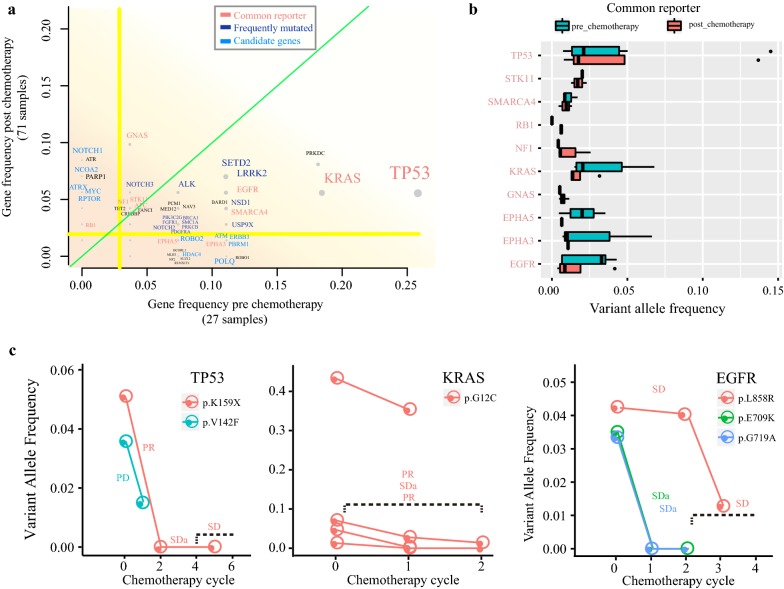
Mutant genes vary during platinum treatment. **a** Landscape of mutant genes during platinum-based chemotherapy. The y-axis represented the gene frequency post chemotherapy, whereas the x-axis represented the gene frequency pre chemotherapy. Each grey dot on behalf of one gene. Gene name with different color denoted its category. Light green line referred x-value equal to y-value. And the yellow lines showed the mean value of gene frequency. Plasma VAFs of common reporter during platinum-based chemotherapy were shown in **b** and **c**

In total, 392 out of 508 (77.2%) cancer-related genes were identified from 98 blood samples (Fig. 4a), including several previously reporter genes: *TP53* (pre vs post: 25.9% vs 5.6%),*KRAS* (pre vs post: 18.5% vs 5.6%), *EGFR* (pre vs post: 11.1% vs 5.6%), *STK11* (pre vs post: 3.7% vs 4.2%), *GNAS* (pre vs post: 3.7% vs 9.9%). Of these common reporter genes, some non-silent protein-coding mutations were observed both pre- and post- chemotherapy (Fig. 4b), showing the important role of these genes in LUAD. We found that VAFs of some driver mutations decreased following platinum treatment, and some hotspot mutations, such as *EGFR(L858R)*, *KRAS* (p.G12C), were still present post chemotherapy(Fig. 4c). Besides reporter genes, chromatin modifying gene *SETD2* was also commonly mutated (pre vs post: 11.1% vs 7%), as well as *NSD1* [25] (pre vs post: 11.1% vs 4.2%), *LRRK2* (pre vs post: 11.1% vs 7%) and ALK (pre vs post: 7.4% vs 5.6%). Mutations in genes *NOTCH1*, *NCOA2*, *MYC*, *ATRX* and *RPTOR* were significantly increased after platinum chemotherapy in the plasma samples (Additional file 7: Figure S3A), whereas few genes were almost absent after treatment, such as *HDAC4, USP9X* and *POLQ* (Additional file 7: Figure S3B), suggesting that neoplastic cells carrying a specific set of somatic mutations were sensitive to platinum treatment. Nonetheless, persistence of genetic mutations like *TP53* (p.V41L), *MYC* (p.Q48H), *ALK* (p.V467L), *ALOX12B* (p.I672 M) and *CASP8* (p.S301Y) were still observed during platinum treatment (Additional file 7: Figure S3C), showing a link between insensitivity to platinum-based chemotherapy with these base substitutions. Notably, a novel cancer gene *ROBO2* (Roundabout Guidance Receptor 2), which plays important roles in apoptosis, motility, angiogenesis and invasion of cancer cells [26, 27], were also mutated in a set of patients (pre vs post: 7.4% vs 1.4%) (Additional file 7: Figure S3D). Moreover, mRNA expression of *ROBO2* were significantly decreased in TCGA cohort [28] (p < 0.001). Three independent cohorts showed the variants of *ROBO2* (Additional file 8: Table S5). These findings suggest that *ROBO2* may play an important role in LUAD tumorigenesis.

### Key pathway involved in chemotherapy and therapeutically targetable driver genes

**Fig. 5:**
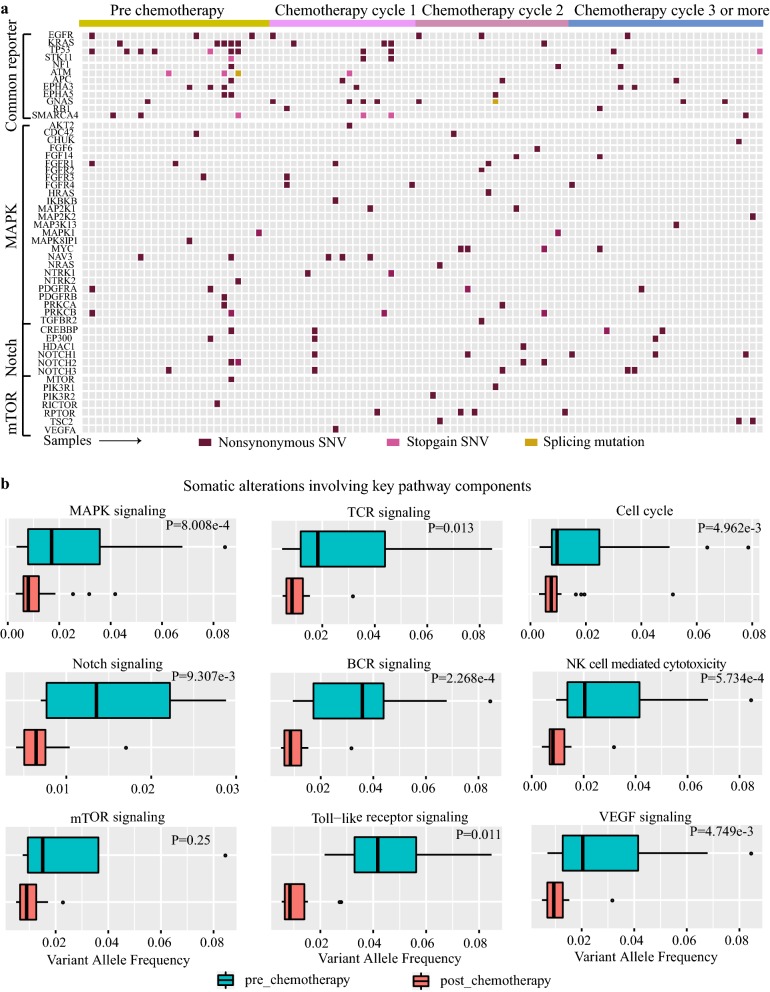
Landscape of potentially clinically actionable variants in plasma of LUAD. **a** Landscape of non-silent mutations of common reporter genes and three important pathway genes during chemotherapy. The y-axis represented the gene, whereas x-axis on behalf of samples. Different color showed different mutant type of non-silent mutations. **b** Plasma VAFs of key pathways during platinum-based chemotherapy

To further understand the genetic dynamics process, we examined the distribution of mutations across Kyoto Encyclopedia of Genes and Genomes (KEGG) pathways. 84 point mutations in 30 genes, including EGF/FGF family members and NTRK receptor families, were found in the MAPK pathway (Fig. 5a). Notably, 52% (51/98) samples carried one or more mutations activated the MAPK pathway, implying this pathway would have a pivotal role in LUAD (Fig. 5a). Moreover, we found that many variants in this pathway presented with lower allele frequencies in plasma samples post chemotherapy (p < 0.001; Fig. 5b), suggesting that those somatic mutations in genes within the MAPK pathway would under the influence of platinum-based chemotherapy. We also discovered seven other pathways with VAFs of their mutations significantly changed during platinum treatment, including Notch signaling, TCR/BCR signaling, cell cycle, VEGF signaling, Toll-like receptor signaling and NK cell mediated cytotoxicity (Fig. 5b). In addition, mTOR pathway components were mutated in 10 genes and in more than 16.3% of tumors. Of these mutations, VAFs didn’t have a prominent change (p = 0.25), showing activation of mTOR pathway through somatic mutations contribute less to platinum-combination chemotherapy in advanced LUAD.

91 genomic variations within 47 genes were detected in 56 plasma tumors from 26 patients based on the TARGET database [29] (Additional file 9: Figure S4A). We found that 50% of patients carried potential therapeutic targets both pre and post-chemotherapy, and there exists some potential therapeutic targets presented in 28.1% (9/32) of patients only after chemotherapy. Although some drug mutations were eliminated, there still has a set of genes for which somatic alterations have therapeutic or prognostic implications, such as *ALK*, *EGFR*, *CDK12* and so on (Additional file 9: Figure S4B). Notably, mutations participating in activating the ERK, PI3K, MTOR pathway were frequent, with 35 alterations in 28 cases occurring in 13 of 17 evaluated genes. Of which, *EGFR* was the most frequently mutated gene in LUAD (7.14% of tumors), following by *ALK* (6.12% of tumors) (Additional file 9: Figure S4C). Based on the CIViC database [30], we identified an original *EGFR*-activating mutation (S768I) in one patient, which can be targeted by Erlotinib and Gefitinib. Simultaneously, *KRAS* G12C mutation was detected in four patients shown to be actionable by ARS-853, *EGFR* Inhibitor, Docetaxel, and Selumetinib (AZD6244). Taken together, these data suggest that ctDNA analysis for LUAD patients can yield significant clinical relevance and detect potentially targetable genes in patients, which can provide guidance for individualized therapy during chemotherapy.

### Relationship of clinicopathological features with chemotherapeutic response and prognosis

**Fig. 6:**
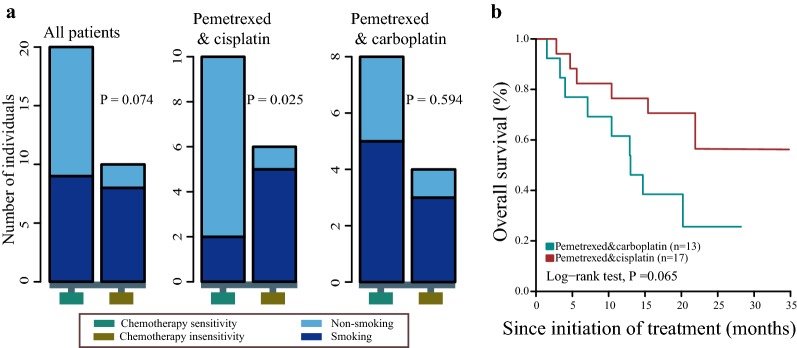
Smoking associated with chemotherapy response. **a** Relationships between smoking and chemotherapy response with different therapeutic plans were shown in the panel. The y-axis represented the number of patients. Vertical columns correspond to chemotherapy sensitivity (aquamarine4) and chemotherapy insensitivity (goldenrod4). **b** Overall survival analysis with different therapeutic regimens

Next, we examined the differences of clinical features between chemotherapy sensitive versus insensitive populations, the result demonstrated that more smokers tend to exist in insensitive group (p = 0.074; Fig. 6a). Interestingly, this association between smoking status and treatment efficacy was not found in patients treated with pemetrexed and carboplatin(p = 0.594), but existed in patients who treated with pemetrexed combined with cisplatin(p < 0.05) Above results suggest that smoking may affect the efficacy of platinum chemotherapy, especially pemetrexed combined with cisplatin treatment. Moreover, the survival analysis revealed that patients treated with pemetrexed and carboplatin got longer survival than pemetrexed combined with cisplatin, though with no statistical significance. (Figure 6b).After first line chemotherapy failure, 37.5%(12/32) patients received TKI treatment, the multivariable analysis showed TKI therapy was significantly associated with better outcome, which was significant independent of other factors (Additional file 10: Figure S5).

## Discussion

This study was designed to explore the dynamic genetic alterations during first line platinum-based doublet chemotherapy in advanced NSCLC patients, and investigate the potential genomic variations associated with clinical response based on 508 cancer-related gene assessments. We explored the great potential of ctDNA in cancer prognostic prediction and depicted the genetic spectrum under chemotherapy. Mutation burden and some key mutations were found decreased following chemotherapy. Several critical pathways and potential drug targets were identified, which might provide guidance of individualized lung cancer therapy.

In fact, ctDNA is convenient to obtain and less risk to patients compared with tissue biopsy. Theoretically, ctDNA may carry more information on the entire tumor regardless of tumor heterogeneity caused by sampling of a single site [31]. CtDNA has been demonstrated to be a potential material for tumor early detection and efficacy monitoring, consequently, FDA has approved several companion diagnostic devices based on blood testing. This study provides that ctDNA assessments could be used to reliably monitor and correlated with clinical outcome to explore a potential technique of cancer treatment. Since analytical validity is critical to the assessment of clinical significance [32], we validated the potential mutations detected in both blood and matched tissues of two patient before treatment. We found that the concordance rates of mutation between blood and tissue were 32.3% and 20%, respectively, which was within a range of 15%-94% in previous report [33].

We noticed that mutation burden was decreased following cycles of chemotherapy, and patients harboring more non-silent mutations showed better response Tumor mutation burden (TMB) was known as related to the efficacy of immune checkpoint inhibitors [34], however, one recent report suggested that TMB may be a predictive marker of chemotherapy response [35]. Our results gave a clue that chemotherapy has an impact on mutation burden, and mutation burden could be a potential marker of clinical response, which increased our understanding of mechanism of chemotherapy and the association between genomic alteration with chemotherapy. Among mutations altered following chemotherapy, C>G substitutions were significantly decreased, and C>A point-mutation was also associated with chemotherapy efficacy (Additional file 11: Table S6).

Furthermore, some common driver genes, including *TP53* (p.K159X) and *EGFR* (p.E709 K and p.G719A) were no longer detected after therapy, indicating these mutations may contribute to sensitivity to chemotherapy, although the biological behavior of these mutations was not fully understood, our study demonstrates their potential effects on chemotherapy and improved our knowledge to lung adenocarcinoma mutation landscape. *TP53* mutation can elicit oncogenic activities besides the loss of tumor suppression function [36], previous studies have explored the predictive role of *TP53* in NSCLC with chemotherapy treatment, but the results were inconsistent. In a 253-patient study, the presence of *TP53* mutation showed as an approval factor in response from chemotherapy [37], while a study with 35 patients indicated that mutant *TP53* associated with resistance to chemotherapy [38], another report involving 524 patients found that no correlation of *TP53* mutation with clinical chemotherapy responses [39]. Further investigations are needed to improve assessment of the prognostic value of *TP53* in chemotherapy treatment. *ROBO2* gene was considered as a tumor-suppressor gene in multiple cancers [24, 40, 41], but this effect could be weakened by mutation [26]. Another study found that mutation in the fibronectin and intracellular region of ROBO may significantly affect the function, and further facilitate disease progression and confer a worse clinical outcome. *ROBO2* mutation may disrupt ROBO signaling, and cause cell growth imbalance and apoptosis, which further lead to progression and poorer prognosis. Our results indicate that *ROBO2* may be a potential target in the treatment of NSCLC patients.

Moreover, besides gene mutation, several pathways were associated with chemotherapy treatment, and might be a potential drug target of lung cancer treatment. MAPK pathway comprised of the MAPK/ERK family, Big MAP kinase-1(BMK-1), c-jun N-terminal kinase (JNK), and p38 signal families [42]. It is reported to be a critical pathway to human cancer survival, differentiation and drug resistance, its importance can be differentiated according to the origin of tissue [43]. Driver mutations (such as *EGFR*, *KRAS* and *BRAF*) have been identified in genes downstream of MAPK/ERK pathway. Our results demonstrated that more than half of the tumors harbored MAPK activating mutation in adenocarcinoma, suggesting that MAPK signal pathway is also involved in the regulation of NSCLC chemotherapy. The PI3K/AKT/MTOR signaling pathway also plays a key role in cancer biology, first generation MTOR inhibitors were approved for treatment of multiple cancer types, including renal, breast and some brain cancers [44]. Potential of this pathway target has been identified for NSCLC chemotherapy [45]. Our present study showed no much genomic alteration changes following chemotherapy treatment in PI3K/MTOR pathway, indicating that the combination of MTOR inhibitor and chemotherapy may be an effective therapeutic strategy for NSCLC. These findings underscore the need for further research into the mechanisms and targeted therapy of MAPK and PI3K/MTOR signal pathway in NSCLC. Somatic alterations including *EGFR, ALK, CDK12* were successfully identified, two of which are targetable by currently available drugs, these findings approved the usage of ctDNA for mutation detection and the potential targeted therapy in NSCLC patients receiving chemotherapy.

In addition, we analyzed the role of smoking in lung cancer. Smoking has been accounted for the development of cancer for a long time and correlated with 87% of lung cancer deaths [46]. Our study showed non-smokers are more likely to benefit from Pemetrexed and cisplatin treatment than smokers, but not for Pemetrexed & carboplatin group, moreover, patients treated with Pemetrexed & cisplatin got a minimal survival benefit than Pemetrexed & carboplatin. In multiple analysis, TKI therapy was significant with overall survival, all these impact on survival caused by smoking status/chemotherapy regimen/TKI therapy should be cognizant by the oncologists.

In conclusion, we explored the dynamic genomic changes in ctDNA in advanced NSCLC patients received chemotherapy, the results demonstrated the potential predictive role of mutation burden and a subset of genes, and underscored the need for additional studies to further assess the biological mechanisms of MAPK and PI3K/MTOR pathway in chemotherapy. Moreover, this study gave a clue that non-smokers can better benefit from Pemetrexed and cisplatin treatment than smokers.

## Supplementary information

**Additional file 1: Table S1.** The clinicopathologic features of patients with NSCLC on chemotherapy.

**Additional file 2: Table S2.** The clinicopathological information and blood draw tme point for each patient.

**Additional file 3: Table S3.** Gene list for BGI Oseq-ctDNA.

**Additional file 4: Figure S1.** Sequencing depth and mutations detected in plasma and tumors. (**A**) Boxplot showed the sequencing depth of target region. Horizontal axis represented normal and tumor groups. Vertical axis represented the average coverage. A dot (red) is on behalf of a sample. **(B)** Variant allele frequency of each mutation was shown in the panel with boxplots. Different color represented different chemotherapy cycle with its mean VAF. One dog on behalf of one sample. **(C)** Mutation detecting profile in two validate tissue sample and corresponding plasma sample. Triangle referred to one mutation detected in both tissue sample and plasma sample. Circle referred to one mutation only detected in plasma sample. Diamond referred to one mutation only detected in tissue sample.

**Additional file 5: Figure S2.** Total SNVs detected in plasma. **(A)** The total SNVs of each sample during chemotherapy was shown. The y-axis represented the mutation number. Each bar represented each sample. Each color represented one of the subsets: all detected mutations, coding region or Non-silent mutations. **(B)** The distribution of non-silent SNVs between individuals with chemotherapy sensitivity (PR/SDa) and other (PD/SD). Ligth blue represents these individuals with chemotherapy sensitivity (PR/SDa). **(C)** The distribution of C>G substitutions between individuals with chemotherapy sensitivity (PR/SDa) and other (PD/SD). **(D)** Boxplot showed the C>A substitution subtype comparison of pre-chemotherapy and post-chemotherapy. The y-axis represented the total SNVs. Each box represented each group and each red dot was on behalf of one sample. **(E)** Boxplot showed the comparison of pre-chemotherapy and post-chemotherapy. Box with lightcoral represented nonsmoker and box with medium turquoise represented smoker. Horizontal axis represented the chemotherapy cycles.

**Additional file 6: Table S4.** Concordant mutations between tumor and plasma samples

**Additional file 7: Figure S3.** Predictors of plasma VAFs during platinum-based chemotherapy. Plasma VAFs of mutant genes **(A, B** and **C)** and Gene express of ROBO2**(D)** between tumor and normal in TCGA cohort, and function domain altered in gene ROBO2. Each dot represented one samples. Two truncating mutations (including nonsense, nonstop, frameshift deletion, frameshift insertion and splice site) were indicated with hotpink2 dot, ***p < 0.001, **p < 0.01, *p < 0.05.

**Additional file 8: Table S5.** Smoking associated signatures and platinum-based chemotherapy.

**Additional file 9: Fifure S4.** Landscape of potentially clinically actionable variants in plasma of LUAD. **(A)**Number of individuals for targeting therapy during chemotherapy and mutated frequency of genes. Pre (goldenrod1): patients have potential drug targets only in pre chemotherapy; Pre & Post (grey): patients have potential drug targets in pre and post chemotherapy; Post (indianred): patients have potential drug targets only in post chemotherapy). Each bar represented a category. **(B)** Number of individuals for targeting therapy, each bar represented a gene. **(C)** Variants in genes (rows) that may predict sensitivity to ERK-Signalling, PI3K/MTOR inhibitor. Vertical columns correspond to plasma samples.

**Additional file 10: Figure S5.** The univariate and multivariate analysis of clinical factors to overall survival. Data were calculated by the method of Kaplan and Meier, with log-rank P value.

**Additional file 11: Table S6.** ROBO2 gene variants in different cohorts.

## Data Availability

The primary datasets used and/or analyzed in the current study are available from the corresponding author (Yuankai Shi, syuankai@cicams.ac.cn) upon reasonable request.
