# Supplementary figures and images for: Tracking longitudinal genetic changes of circulating tumor DNA (ctDNA) in advanced Lung adenocarcinoma treated with chemotherapy
Source: J Transl Med. 2019 Oct 10;17:339. doi: 10.1186/s12967-019-2087-9 (PMC6785899; doi:10.1186/s12967-019-2087-9)

A

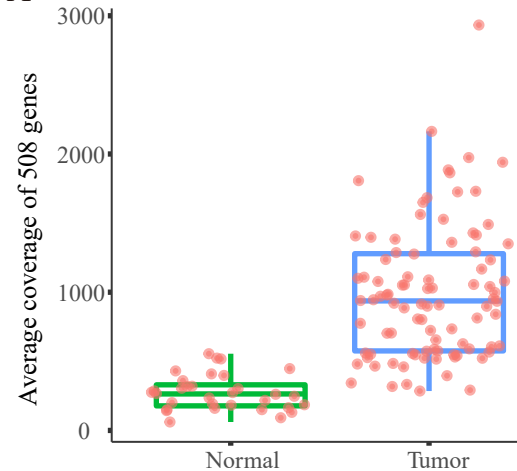

B

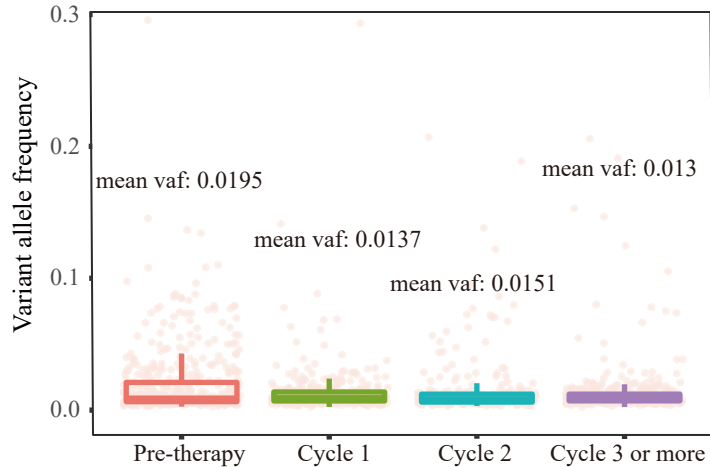

C

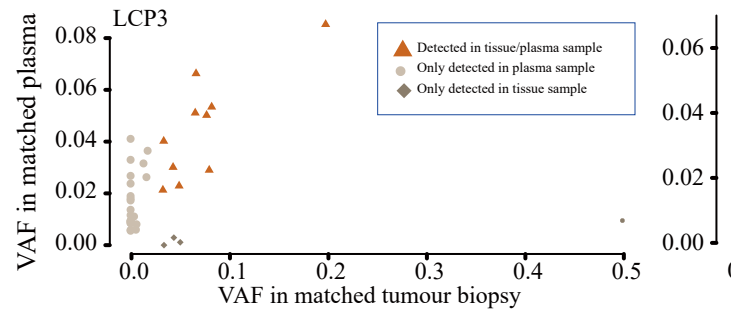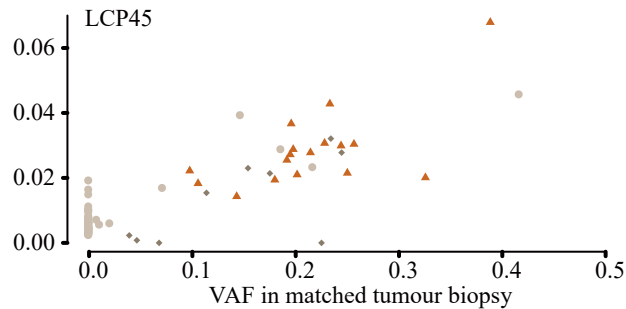

Supplement: Supplementary file 4 — Additional file 4: Figure S1. Sequencing depth and mutations detected in plasma and tumors. (A) Boxplot showed the sequencing depth of target region. Horizontal axis represented normal and tumor groups. Vertical axis represented the average coverage. A dot (red) is on behalf of a sample. (B) Variant allele frequency of each mutation was shown in the panel with boxplots. Different color represented different chemotherapy cycle with its mean VAF. One dog on behalf of one sample. (C) Mutation detecting profile in two validate tissue sample and corresponding plasma sample. Triangle referred to one mutation detected in both tissue sample and plasma sample. Circle referred to one mutation only detected in plasma sample. Diamond referred to one mutation only detected in tissue sample. [file 12967_2019_2087_MOESM4_ESM.pdf]

A

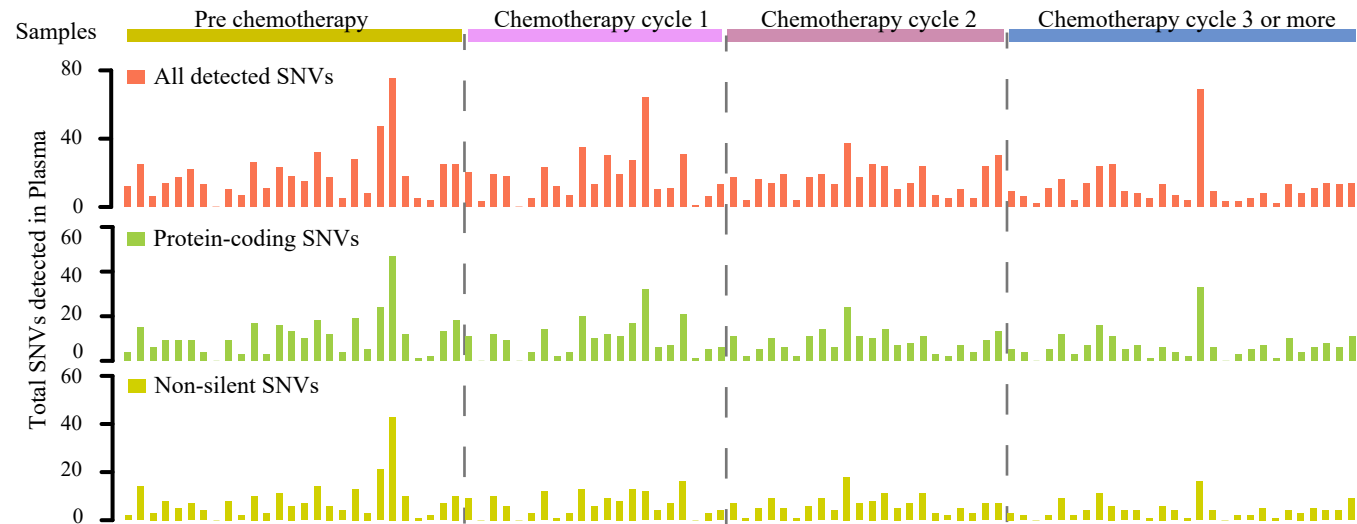

B

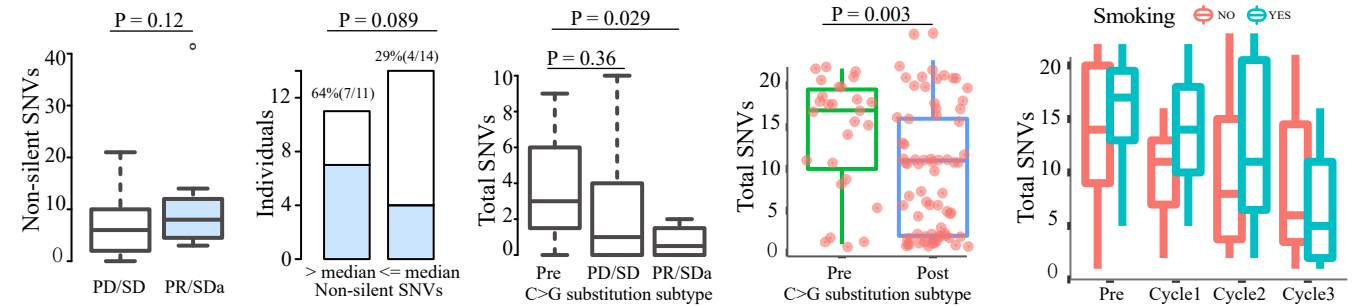

Supplement: Supplementary file 5 — Additional file 5: Figure S2. Total SNVs detected in plasma. (A) The total SNVs of each sample during chemotherapy was shown. The y-axis represented the mutation number. Each bar represented each sample. Each color represented one of the subsets: all detected mutations, coding region or Non-silent mutations. (B) The distribution of non-silent SNVs between individuals with chemotherapy sensitivity (PR/SDa) and other (PD/SD). Ligth blue represents these individuals with chemotherapy sensitivity (PR/SDa). (C) The distribution of C>G substitutions between individuals with chemotherapy sensitivity (PR/SDa) and other (PD/SD). (D) Boxplot showed the C>A substitution subtype comparison of pre-chemotherapy and post-chemotherapy. The y-axis represented the total SNVs. Each box represented each group and each red dot was on behalf of one sample. (E) Boxplot showed the comparison of pre-chemotherapy and post-chemotherapy. Box with lightcoral represented nonsmoker and box with medium turquoise represented smoker. Horizontal axis represented the chemotherapy cycles. [file 12967_2019_2087_MOESM5_ESM.pdf]

A

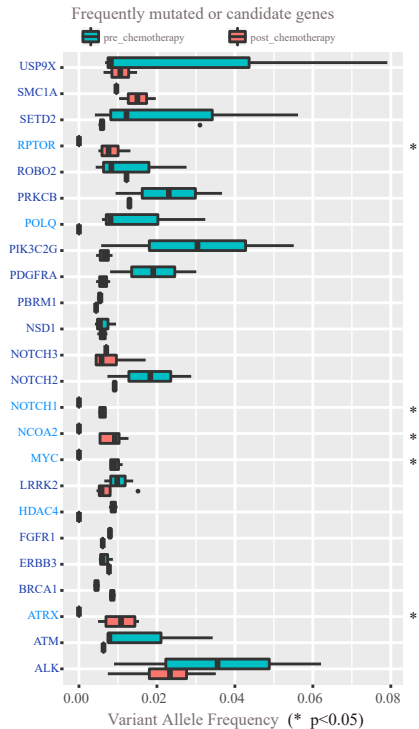

B

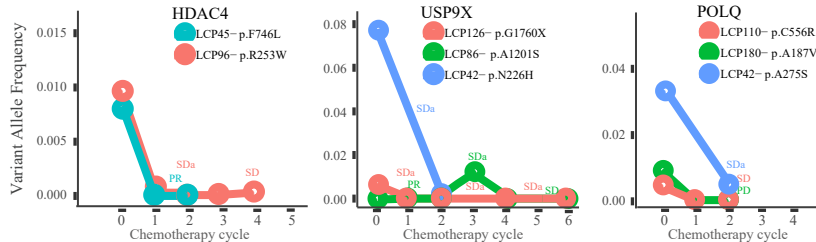

C

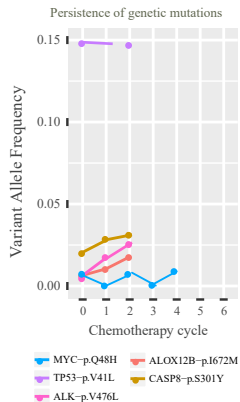

D

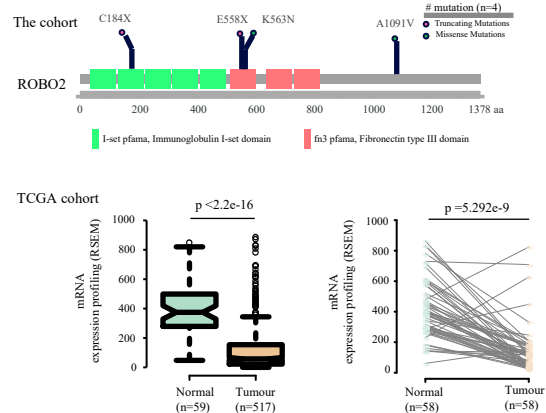

Supplement: Supplementary file 7 — Additional file 7: Figure S3. Predictors of plasma VAFs during platinum-based chemotherapy. Plasma VAFs of mutant genes (A, B and C) and Gene express of ROBO2(D) between tumor and normal in TCGA cohort, and function domain altered in gene ROBO2. Each dot represented one samples. Two truncating mutations (including nonsense, nonstop, frameshift deletion, frameshift insertion and splice site) were indicated with hotpink2 dot, ***p < 0.001, **p < 0.01, *p < 0.05. [file 12967_2019_2087_MOESM7_ESM.pdf]

A

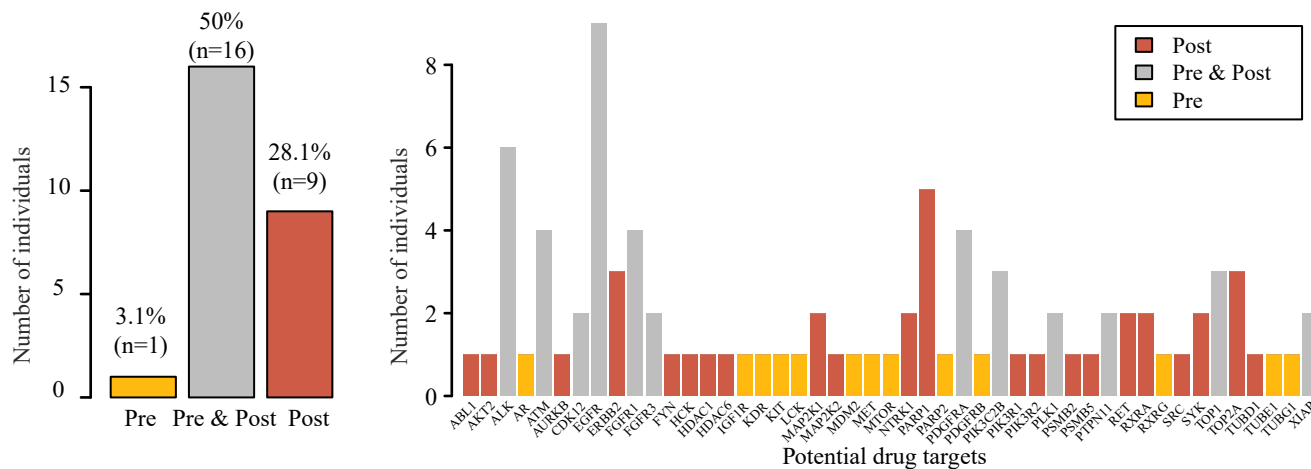

B

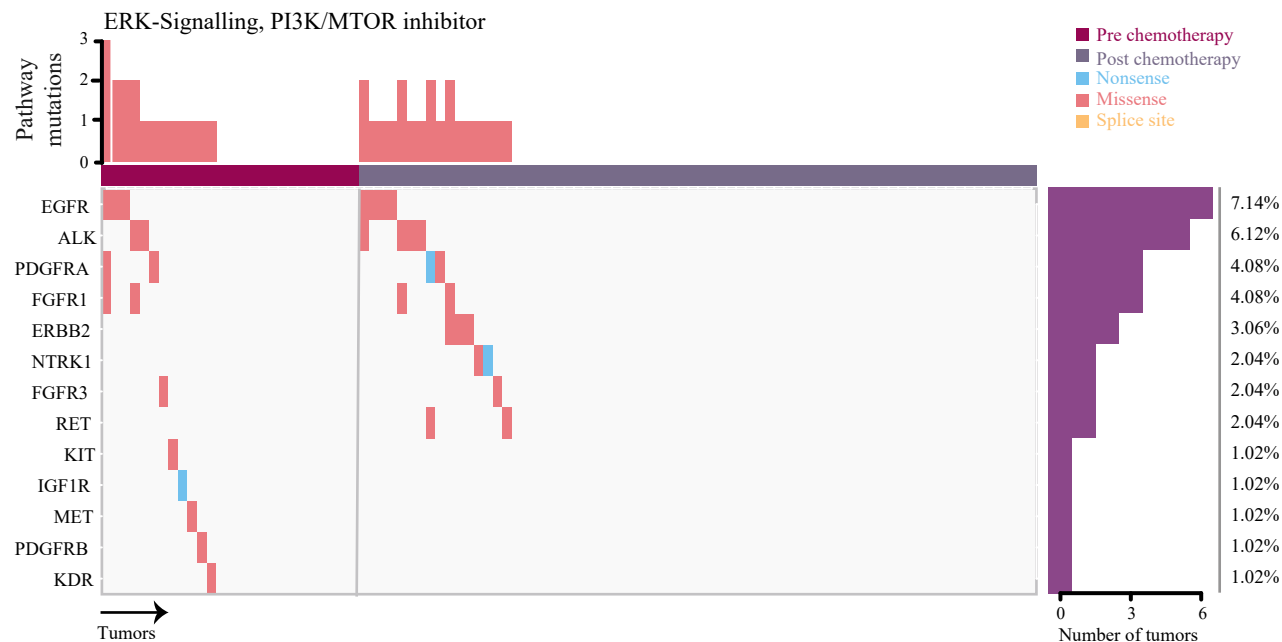

Supplement: Supplementary file 9 — Additional file 9: Fifure S4. Landscape of potentially clinically actionable variants in plasma of LUAD. (A)Number of individuals for targeting therapy during chemotherapy and mutated frequency of genes. Pre (goldenrod1): patients have potential drug targets only in pre chemotherapy; Pre & Post (grey): patients have potential drug targets in pre and post chemotherapy; Post (indianred): patients have potential drug targets only in post chemotherapy). Each bar represented a category. (B) Number of individuals for targeting therapy, each bar represented a gene. (C) Variants in genes (rows) that may predict sensitivity to ERK-Signalling, PI3K/MTOR inhibitor. Vertical columns correspond to plasma samples. [file 12967_2019_2087_MOESM9_ESM.pdf]
