# Supplementary material for: Tracking longitudinal genetic changes of circulating tumor DNA (ctDNA) in advanced Lung adenocarcinoma treated with chemotherapy
Source: J Transl Med. 2019 Oct 10;17:339. doi: 10.1186/s12967-019-2087-9 (PMC6785899; doi:10.1186/s12967-019-2087-9)

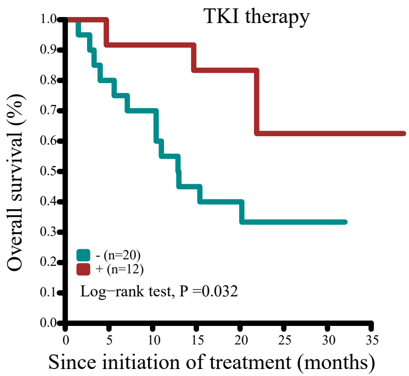

Univariate analysis

Variable	HR (95% CI)	P−value
Age	1.03 (0.972 ~ 1.09)	0.307
Gender (male vs. female)	0.651 (0.221 ~ 1.91)	0.436
stage (III vs. IV)	3.55 (0.794 ~ 15.9)	0.0973
smoking_history (smoking vs. non-smoking)	1.45 (0.516 ~ 4.1)	0.479
pleural_effusion (yes vs. no)	1.55 (0.548 ~ 4.37)	0.41
chemotherapy (carboplatin vs. cisplatin)	0.39 (0.138 ~ 1.1)	0.0755
TKI (yes vs. no)	0.259 (0.0728 ~ 0.924)	0.0374

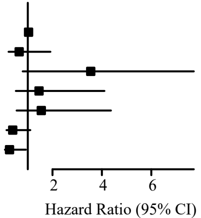

Multivariate analysis

Variable	HR (95% CI)	P−value
Age	1.03 (0.95 ~ 1.12)	0.449
Gender (male vs. female)	0.466 (0.0309 ~ 7.04)	0.582
stage (III vs. IV)	5.37 (0.86 ~ 33.6)	0.0721
smoking_history (smoking vs. non-smoking)	1.01 (0.0644 ~ 15.7)	0.997
pleural_effusion (yes vs. no)	2.11 (0.532 ~ 8.37)	0.289
chemotherapy (carboplatin vs. cisplatin)	0.903 (0.222 ~ 3.67)	0.887
TKI (yes vs. no)	0.149 (0.0357 ~ 0.619)	0.00881

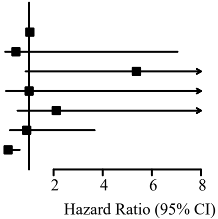

Supplement: Supplementary file 10 — Additional file 10: Figure S5. The univariate and multivariate analysis of clinical factors to overall survival. Data were calculated by the method of Kaplan and Meier, with log-rank P value. [file 12967_2019_2087_MOESM10_ESM.pdf]
